# Musculoskeletal Injuries in Capoeira Athletes: An Epidemiological Study

**DOI:** 10.3390/healthcare11141978

**Published:** 2023-07-08

**Authors:** Beatriz Minghelli

**Affiliations:** 1 Escola Superior de Saúde Jean Piaget Algarve, Instituto Piaget, 8300-025 Silves, Portugal; beatriz.minghelli@ipiaget.pt; Tel.: +351-282-440-170; 2 KinesioLab—Research Unit in Human Movement, Av. João Paulo II, lote 544, 2° andar, 1950-157 Lisboa, Portugal

**Keywords:** incidence, capoeira, injuries

## Abstract

Capoeira involves fighting movements, turns, acrobatic jumps, and repeated movements, which can lead to injury. This study determined the incidence of injuries in capoeira athletes and analyzed the associated factors. The sample included 157 capoeira athletes, 94 (59.9%) of which were males aged 8–67 years. A questionnaire was administered; in the results, 95 (60.5%) athletes suffered injury during their entire capoeira practice, totaling 218 injuries, and 48 (30.6%) athletes had incurred an injury in the previous year, totaling 81 injuries. There were 0.85 injuries per 1000 h of capoeira training. The most common injuries were sprains (19.23%) and muscle bruises (14.10%), which were located in the ankles (20.51%) and knees (16.67%). Falls (24.36%) and repetitive movements (15.38%) were the most prevalent injury mechanisms. Male athletes presented a higher risk of sustaining capoeira-related injuries than women (odds ratio = 2.19; 95% CI: 1.05–4.61; *p* = 0.037). Individuals who trained equal to or more than three times per week were more at risk by 0.44 (*p* ≤ 0.001) than those who trained up to two times per week. This study showed a high prevalence of injuries in this sample. Sex (male) and a training frequency that was equal to or greater than three times per week were the associated risk factors. The data obtained can be used to create specific training programs for preventing injuries.

## 1. Introduction

Capoeira is a Brazilian cultural manifestation that combines different elements such as fighting, dancing, art, folklore, leisure activities, games, and sports [1,2,3,4]. Capoeira is a fight in terms of being an instrument of combat and self-defense. It is dance and art through the diversity of its gestures and musicality. It is folklore as a representation of Afro-Brazilian culture. It is partly leisure and game as a way of promoting socialization and living together in open spaces. Lastly, it is a sport because it involves specific training (physical, technical, and tactical training) [5].

Capoeira is characterized as a game/fight of corporal dexterity that is practiced in pairs, with the people forming a circle, and involves dancing and acrobatic movements [1,6]. Capoeira, although called a martial art, is a dance and game [7,8], and its practitioners (capoeiristas) are players and not fighters; the verb used is “to play”, not the verb “to fight” [8]. This game takes place under the sound of musical instruments, namely atabaques, tambourines, and berimbaus [9].

Capoeira began during the period of slavery, emerging as a process of struggle against social and racial prejudices. There are several hypotheses that explain the emergence of capoeira, the most acceptable being that it was created in Brazil by slaves [3].

Capoeira is a form of fighting that would have been disguised as a dance to elude and circumvent the ban on its practice by overseers and plantation owners during the period of slavery. After the abolition of slavery, free black labor encountered serious obstacles to integrate into the economy due to centuries of slavery tradition; thus, capoeiristas lived in marginality. They committed crimes, and capoeira was seen as something that was practiced by marginal persons, having been prohibited by law by the Penal Code at the beginning of the Republic by decree nº 487 in October 1890 [3,10]. Only in 1930 was this law revoked, and the first gymnasiums for teaching capoeira opened in Salvador [3].

Capoeira became a competitive sport, according to the resolution of the National Sports Council, in 1972 [9]. It was an art that slowly gained the connotation of a national sport, an art that emerged from marginalization and prohibition by the penal code [10]; the internationalization of capoeira began in the 1970s through artistic shows and was seen as something exotic [11]. 

Currently, capoeira is widely practiced not only in Brazil, but in more than 150 countries; there are 71 countries that have registered capoeira circles, and they represent an international popular cultural phenomenon of Brazilian culture [2,7,10,12]. It is estimated that there are 8 million capoeira practitioners around the world, with approximately 6 million being in Brazil [13]. Capoeira is a modality that was initially prohibited but is currently recognized as one of the richest and most complete cultural and sporting manifestations, having arisen from the historical and social context experienced by the Brazilian people [1].

In Portugal, capoeira was introduced with Brazilian emigration in the 1980s; in the early 1990s, the first associations of practitioners were created, and the first meetings and capoeira groups were organized [14]. A survey carried out by Falcão (2004, cited by 14) found that there were around 35 Brazilian teachers teaching in Portugal in 2003. In 2010, Carvalho [14] counted 100 teachers, some of which were Portuguese, and also counted 55 capoeira groups, reflecting a growth and consolidation of capoeira in Portugal.

The practice of capoeira develops flexibility, agility, coordination, balance, dexterity, strength, and aerobic endurance [5,6,7,13,15]. In a capoeira circle, several fighting movements occur, such as escapes/dodges; turns; acrobatic jumps; insinuations that focus on the partner; sudden changes in direction; attacks through punches, elbows and headbutts, which are rarely used; and shots that are not routinely blocked but deflected [7,8,16], with physical contact being rare [1,8]. For the practicing of capoeira, no protective materials are used, including helmets, shin guards, gloves, and gum protectors, and capoeira games usually take place with the individuals being barefoot [8].

Maneuvers, such as kicks and dodges; blows; sudden changes in direction; jumps and landings; acrobatics, such as backflips and spins; and repeated movements can lead to a high overload on the musculoskeletal system, making it more susceptible to injuries and pain [4,6,13,15,16,17].

Currently, studies on the epidemiology of injuries in capoeira practitioners are scarce, and most are not representative as they did not define the study population or calculate the sample or have other methodological weaknesses. In light of this information, the aim of this study was to determine the epidemiology of musculoskeletal injuries in capoeiristas and verify the associated risk factors.

## 2. Materials and Methods

This was a cross-sectional study that was approved by KinesioLab—Research Unit in Human Movement.

All participants were informed about the objectives of the study and that they could withdraw from it without any type of prejudice. The anonymity of individual responses was ensured because the results were obtained on a global scale and were not associated with individual responses; furthermore, the athletes’ names were not associated with the given responses. In the case of minors, parents and/or coaches authorized participation in the study.

### 2.1. Participants

The population consisted of capoeira athletes of all sexes aged 10 years or over who were present (not necessarily those who participated in these competitions) in the “Copa Muzenza Capoeira” competition (116 athletes enrolled), which was held in Portugal, and in the 11th Open World Championship of Capoeira Muzenza (100 athletes enrolled in the advanced category), held in Brazil. The word “athlete” is used to refer to the people who practiced capoeira.

The inclusion criteria cumulatively involved athletes who practiced this modality for a period of equal to or greater than 1 year, those who performed at a training frequency of at least twice per week, and those who agreed to freely participate in the study.

The sampling method was non-probabilistic, as all athletes who participated in this championship and were eligible to participate in the study were asked if they wanted to participate.

The sample calculation took into account the number of athletes enrolled in the competitions “Copa Muzenza Capoeira” and the 11th Open World Championship of Capoeira Muzenza; we considered a population of 216 athletes, an estimated prevalence of 65% injuries (as reported in national studies) [4,13,15,16,18], a confidence interval of 95%, and an error margin of 5%, and we set an *n* number of 134 athletes as the representative sample.

### 2.2. Measuring Instruments

The measurement instrument used consisted of a questionnaire that was divided into two parts: a sociodemographic characterization of the population and aspects related to the modality and questions about the occurrence of injuries.

As there were no known validated questionnaires on injuries in capoeira, the questionnaire was prepared and evaluated by a group of three specialists with different backgrounds (a physiotherapist with a PhD in epidemiology; a capoeira trainer who was a practitioner for 19 years, the European champion in 2010, 2011, 2012, and 2016, and the World Champion in 2017; a master of capoeira 5th degree by the Muzenza Group of Portugal, who was coordinator of the Muzenza Group of Capoeira in Europe and had a degree in physical education).

The questionnaire was submitted to a pre-test, which was applied to 10 athletes in order to verify the existence of doubts and calculate the time needed to complete the questionnaire (average time: 5.1 min).

The data were collected in person by researchers during the competition “Copa Muzenza Capoeira”, which took place in Seixal (a Portuguese city belonging to the district of Setúbal and the Metropolitan Area of Lisbon) on 3 December 2022 and was disclosed through a QR code during the 11th Open World Championship of Capoeira Muzenza held in Curitiba (Paraná, Brazil) on 28 and 29 January 2023.

The first part of the questionnaire involved the following questions: age, sex, years of practice of the modality, regularity of training per week, weekly training hours, participation in championships, capoeira group affiliation, country where the participant practiced capoeira, graduation, participation in some other sport modality at least twice per week, whether participants warmed up before training/competition, whether participants cooled down when training/competition ended, and the type of capoeira practiced (regional, Angola, contemporary).

The second part of the questionnaire asked about the presence of injuries that occurred with the practice of capoeira in the following periods: throughout the practice, when filling out the questionnaire, and in the last 12 months. If there were injuries, the athletes were asked to mention the number of injuries suffered in each of these moments.

Athletes who had suffered an injury in the last 12 months were asked to continue filling out the questionnaire regarding the characteristics of the injury, such as the type; location; time of occurrence; whether treatment was carried out and, if so, the type of treatment applied; the injury mechanism, if believed that the injury had been caused by practicing on uneven ground or a maneuver had caused the injury; the downtime (lost time from training); and the current injury situation. Respondents were allowed to specify the characteristics of a maximum of three injuries (those considered more serious and/or that required more time for recovery).

An injury was defined as any condition or symptom that occurred as a result of capoeira practice (training and competition) and had at least one of the following effects: the practitioner had to interrupt training/competition for at least one day; the practitioner did not have to stop the sport, but had to modify it (the participant participated with fewer hours of practice or training, under a lower intensity of effort, or was less able to perform certain gestures or movements/techniques); or the professional sought advice or treatment from healthcare professionals to treat the condition or symptom [19].

### 2.3. Data Analysis

The software used to perform the statistical analysis of the data was the Statistical Package for Social Sciences (SPSS), version 28.0.

Descriptive statistics were performed, and binary logistic regressions (Enter methods) were applied to test the influence of the variables used in this study on the injury presence. The statistical significance level was established at 0.05.

To determine the injury proportion, the total number of participants who had at least one injury in the last 12 months was divided by the total number of athletes. The injury rate value refers to the total number of injuries divided by the total time the athlete is exposed to risk (defined 1000 h). The total time of injury risk was calculated by multiplying the average total hours of training by the frequency of training, both over a period of 1 week, and this value was multiplied by 12 months (52 weeks). The average number of injuries per athlete was calculated by dividing the total number of injuries by the total sample number of athletes. The average injuries per injured athlete was calculated by dividing the total injuries number by the total number of injured athletes [20].

## 3. Results

The sample comprised 157 capoeira athletes (fulfilling the representativeness of the study population), with the majority (94; 59.9%) being male and 62 (39.5%) being female athletes; the sample was aged between 10 and 67 years (26.10 ± 15.17 years).

Regarding training characteristics, the weekly frequency ranged from 1 to 12 times (including bi-daily training) (2.82 ± 1.10), with a weekly workload ranging between 1 and 40 h (4.19 ± 3.79); the athletes had between 1 and 54 years of capoeira practice (11.58 ± 11.91).

Most of the sample (129; 82.2%) trained in a capoeira group in Portugal, and 28 (17.8%) athletes trained in Brazil. The vast majority (122; 77.7%) of athletes trained in the Muzenza capoeira group, 4 (2.5%) trained in Associação Dandá, and 15 (9.6%) trained in Agbara. The following capoeira groups were represented by one (0.6%) athlete each: Artemandinga, Associação 100% capoeira, Associação de Capoeira Engenho, Associação Renovar Capoeira Arte e Cultura, Capoeira Angola Palmares, Capoeira Brasil, Capoeira Camboatá, Centro, Centro Cultural Ilê de Bamba, Centro Cultural Rucungo Capoeira, CIA Capoeira, Escola Indigena Tapeba Capoeira, Esporão, Grupo Capoeira Brasil, and Real Sport Club e Vitoria.

Regarding graduation, 56 (35.67%) athletes had received raw graduation, 16 (10.19%) had gray, 11 (7.01%) had yellow, 9 (5.73%) had orange, 22 (14.01%) had green, 3 (1.91%) had monitor, 5 (3.18%) had instructor, 14 (8.92%) had professor, 8 (5.10%) had counter master, and 11 (7.01%) had master; two (1.27%) respondents did not properly respond to this question. Color graduations were grouped in the first color of graduation; for example, red-green was categorized as green. One hundred nineteen (75.8%) of the athletes participated in capoeira competitions, and thirty-eight (24.2%) had never participated. Most athletes (154; 98.1%) reported that they performed some type of warm-up before starting training or competition, and only three (1.9%) athletes reported that they did not. Regarding cooling down, 109 (69.4%) athletes said that they did so and 48 (30.6%) said that they did not.

When asked about the practice of another sport with a frequency of at least twice per week, 77 (49%) athletes answered affirmatively and 80 (51%) said that they did not practice another sport.

Regarding the type of capoeira practiced, 99 (63.1%) reported that they practiced contemporary capoeira, 37 (23.6%) practiced regional capoeira, 9 (5.7%) practiced Angola capoeira, 7 (4.5%) athletes practiced the three types, 3 (1.9%) athletes practiced regional and Angola, and 2 (1.3%) athletes practiced regional and contemporary.

Ninety-five (60.5%) athletes reported having suffered some kind of injury during the entirety of their capoeira practice, with 35 (36.84%) athletes reporting suffering 1 injury, 22 (23.16%) athletes suffering 2 injuries, 13 (13.68%) athletes suffering 3 injuries, and 25 (26.32%) athletes suffering 4 or more injuries, totaling 218 injuries.

When filling out the questionnaire, 36 (22.9%) athletes were already injured. In the last 12 months, 48 (30.6%) athletes reported having suffered an injury, with 30 (62.50%) athletes reporting having suffered 1 injury, 6 (12.50%) athletes having suffered 2 injuries, 9 (18.75%) athletes having suffered 3 injuries, and 3 (6.25%) athletes having suffered 4 or more injuries, totaling 81 injuries.

The value of injury proportion was 0.31 (CI 95%: 0.26–0.36), and the injury rate was 0.85 injuries per 1000 h of capoeira training. The average number of injuries per athlete was 0.52. The average injuries per injured athlete was 1.69.

**Figure 1:**
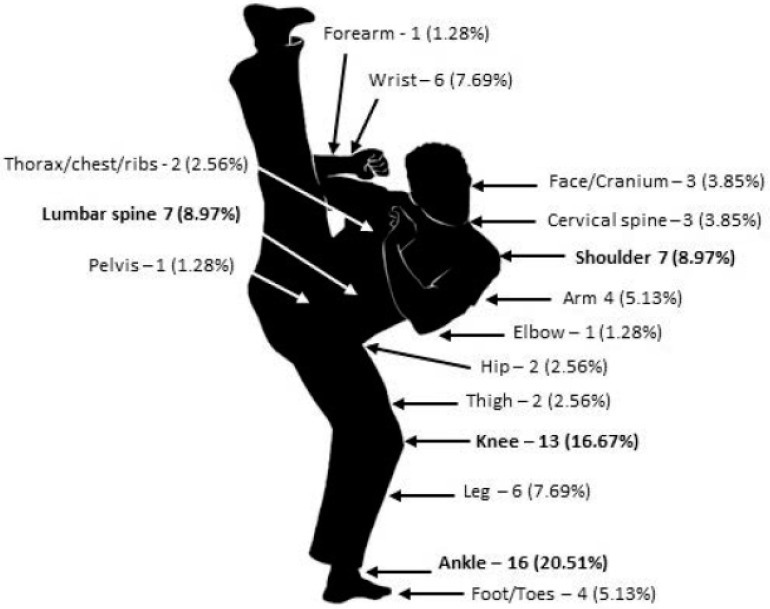
Injured anatomical sites.

Figure 1 shows the relative and absolute frequency of the anatomical sites that presented lesions in the 12-month period.

The values obtained on the type and anatomical site of the measured injuries are presented in Table 1. The number of injuries shown in the table is less than the total number of injuries in the 12-month period because only a maximum of three injuries were classified per athlete.

Most injuries occurred during training sessions/circles inside the gym (58; 74.36%); the remaining injuries occurred during training sessions/circles outside the gym (8; 10.26%), during competitions (6; 7.69%), during warming up before training/competition (3; 3.85%), and during training/outside the gym (on the street) (3; 3.85%).

Table 2 presents the injury mechanisms that were referred to by the athletes.

Most athletes (69; 88.46%) said that they did not think their injury was caused by practicing on uneven ground, whereas 9 (11.54%) athletes agreed that their injury was caused by practicing on uneven ground. Table 3 presents the maneuvers that caused most injuries, with the so-called armed maneuvers being the most common.

Regarding treatment, 59 (75.64%) were treated and 19 (24.36%) were not. Most injuries were treated by rest (24; 27.91%), which was followed by physiotherapy (20; 23.26%), medication (18; 20.93%), immobilization (13; 15.12%), osteopathy (5; 5.81%), unconventional therapies (4; 4.65%), and surgery (2; 2.33%). The total number of types of treatments was greater than the number of injuries because some athletes performed more than one type of treatment.

As for the downtime due to the injury, 7 (10.26%) injuries resulted in up to 2 days of inactivity, 11 (15.38%) occupied between 3 and 7 days, 6 (8.97%) occupied between 8 and 14 days, 5 (7.69%) occupied between 15 and 30 days, 14 (19.23%) occupied more than 30 days, and 30 (38.46%) did not lead to any days of inactivity, although the athletes practiced capoeira conditionally.

The current injury data indicated that in most injuries (36; 48.72%), the athletes no longer had pain or other symptoms and were fully recovered; in eight (11.54%) of the injuries, the athletes did not have pain or other symptoms but were still undergoing treatment and/or conditioning in their sports practice. In 11 (16.667%) of the injuries, the athletes had pain or another symptom and were under treatment, and in 16 (23.08%) of the injuries, the athletes had pain or another symptom but were not in treatment.

The relationship between the injury presence in a period of 12 months and the variables analyzed in this study are shown in Table 4. The graduation variable was initially grouped while excluding raw grading; in the second grading variable, this was split into raw grading by itself and all other gradings grouped together. Athletes practicing Capoeira Angola were not considered in this analysis when the types of capoeria were compared, as the number of practitioners of capoeira Angola was very small.

It was found that capoeira athletes who trained equal to or more than three times per week showed a 0.44 higher probability of developing an injury (95% CI: 1.3–3.5; *p* = 0.003) compared with athletes who trained under three times per week.

## 4. Discussion

The data obtained in this study revealed a high prevalence of injuries throughout the practice of capoeira (61%), at the time of data collection (23%), and in the last 12 months (31%). Higher values were verified in the Sanchez et al. study [21] that evaluated a sample of 200 athletes, wherein 47% of the athletes reported an injury at the time of data collection and 88% reported an injury throughout the entire practice; a similar finding was reported by Zucca and Grüninger [13], who evaluated 51 athletes and found that 71% had injuries throughout practice.

Bonfim [15] evaluated 100 students and teachers of capoeira in Fortaleza and found that 44% of the athletes suffered some injury with the practice of capoeira. Neto et al. [16] evaluated 49 capoeiristas from the city of Salvador Bahia and found a prevalence of 48.9% suffering injuries. Andrade [22] evaluated 53 capoeiristas from the Federal District and found athletes who reported having suffered an injury at a rate of 66%. Signoretti et al. [23] evaluated 16 capoeristas in São Paulo and found an injury prevalence of 68.8%. Campos et al. [4] applied the Self-Estimated Functional Inability of Pain questionnaire for athletes (SEFIP-sport) in a sample of 65 capoeira athletes, and the data revealed that 64.6% of them reported pain or discomfort. Freire et al. [18] evaluated 47 capoeiristas, and the data revealed that 70.2% of the interviewees suffered injuries in capoeira. Moraes et al. [17] evaluated 45 capoeira practitioners and verified the presence of pain and/or lumbar discomfort in 36.6% of the individuals. The data collection in all the studies referred to above was carried out in Brazil, and the temporal period of the injuries in all of these studies was not described, making it difficult to compare them with the data obtained from this study.

Capoeira practice requires combined and successive movements, which are executed in all directions; at high speed with constant turns; mostly on one foot, one arm, or on the head, and even in inverted support, as well as throws and acrobatics; and combined with attacks and dodges [1,4,16], which can lead to body imbalance due to possible changes in flexibility, muscle strength, postural balance and/or coordination, subjecting capoeiristas to postural adaptations that can increase the risk of injuries [2].

Neto et al. [16] evaluated 25 capoeira practitioners who practiced Capoeira Angola and 24 who practiced regional capoeira and found a prevalence of injuries of 28% and 70.8%, respectively. In this study, only 6% of the sample practiced Capoeira Angola, and they were excluded from the logistic regression statistical analysis; we have only analyzed individuals who practiced regional, and the data were not statistically significant. Regional capoeira uses high-speed movements and incorporates techniques from other fights, including grappling movements, kicks, and hand strikes, as in boxing [13,16]; acrobatic movements (jumps) from Olympic gymnastics [17]; and quick kicks, with strikes being performed on the ground and often upside down. Contemporary capoeira encompasses characteristics of regional and Angola capoeira; the latter involves slower strokes with the execution of movements that require one’s hands to be on the ground, legs to be raised low and flexed, and torso and waist to be low [13,16].

Regarding the most frequent anatomical sites of injury, the data from this study revealed that the sites were the ankles (21%), knees (17%), shoulders (9%), and lumbar spines (9%). Similar data were found in the study by Neto et al. [16] in which the most affected sites were the ankles, followed by the knees and shoulders. The study by Andrade [22] reported that 14% of injuries were located in the knees, 14% were in the shoulders, 13% were in the ankles, and 11% were in the spines. In the study by Sanchez et al. [21], the authors presented the knees (21%), ankles (16%), and shoulders (13%) as the anatomical sites that suffered the most injuries.

Carrying out turning movements, usually under one limb (armada, rabo de arraia, chapa giratória), with sudden changes in direction that are associated with movement speed and acrobatics require great sensorimotor stability of the ankle and knee [24] and a recruiting of the neuromuscular spindles; if the athlete does not have good dynamic stability at a muscular level, injuries may occur due to stretching of muscle, tendon, and ligament structures or as a result of a fall. Learning a new gesture can also cause injuries due to body instability if improperly performed without concentration while performing the new movements [8,24]. In our study, the most common injuries located in the ankle were sprains; ligament injuries; muscle bruises; tendinopathy, probably from repeated efforts; and luxation.

Regarding the knee, the pain can be directly linked to the overload imposed on this joint by the sudden change in direction, hyperextension caused by the execution of kicks and dodges, and jumps and landings that are common in the practice of capoeira, which can accentuate the frictional forces and make this region more susceptible to injury and pain [4]. The most frequent types of knee injuries obtained in our study were sprains; meniscal injuries; bruised and sprained muscles; cartilage damage, possibly from repeated efforts; ligament injuries; and osteoarthritis. Shoulder injuries can be justified by performing low strokes that support the body weight on the upper limbs [13] in addition to performing other movements where the body is supported by the hands or even by one of the hands; i.e., on the shoulders, the main injury factor is muscle instability. For example, the kidney fall and suspended bridge maneuvers require great stability [24]. The most common shoulder injuries in our study were ligament injuries; tendinopathy, probably from repeated efforts; and muscle bruises. The injuries in the lower back can be caused by some blows and acrobatics movements, such as mortals, screws, falls, and flourishes, which can cause compressive force due to the impact on the ground [4].

The body regions with the highest reports of pain and disability in the study of Campos et al. [4] were the knees (22%), lower backs (20%), and wrist/hands (17%). Signoretti et al. [23] revealed that the most affected segments were the ankles and feet (31.25%). Freire et al. [18] found that the knees (36.2%), backs (25.5%), shoulders, wrists, ankles, and feet (23.4%) were the most prevalent sites. Zucca and Grüninger [13] also found that the knees were the most injured site (56%), followed by the feet (35%), shoulders (28%), lumbar spine (25%), wrists (23%), and ankles (18%). In the study by Bonfim [15], the main injuries found in the practice of capoeira were located in the knees (17%) and spines (17%).

The most common type of injury was sprains (19%), followed by muscle bruises (14%) and ligament injuries (12%). Sprain can be justified by the athletes carrying out several maneuvers in which there is unipodal support and by the sudden changes in direction, which are associated with the speed of movement and the practice of capoeira requires. We believe that most sprains were not caused by practicing on an uneven floor because most athletes said that they did not think that their injury was caused by practicing on uneven ground and because most of the injuries occurred during training sessions/circles inside the gym. Although capoeira is not a contact sport, it can occur when the other practitioner is unable to dodge the opponent’s blow, and it is in this way that muscle contusion injuries occur. Ligament injuries occur when the joint is subjected to rotational movements, especially when it is subjected to an increased body load, as in the case of unipodal supports and maneuvers that occur with the support of the hands.

Neto et al. [16] revealed that the most frequent types of injuries in athletes were luxation and muscle injury, followed by sprains and fractures. In the study of Sanchez et al. [21], the most frequent injuries were ligament injuries (15.1%), sprains (14.8%) and luxations (11.7%) and in the study by Freire et al. [18] were muscle injuries (38.3%) and fractures (17%).

The main mechanisms that caused injuries in the athletes who constituted the sample in this study were falls, repetitive movements, acrobatic jumps, and contact with another athlete. Similar data were found in the study by Sanchez et al. [21], who presented overuse (21.2%), acrobatics (15.5%), and turns such as armed, jaw, and half moon (12.7%) as the injury mechanisms that were most reported by athletes. Zucca and Grüninger [13] referred to acrobatics as the most common injury mechanism (49.1%), followed by turns (35%) and falls (28%).

A fall may possibly occur during the learning of new gestures, due to the athlete’s lack of concentration when performing the maneuver, due to execution errors, and/or due to muscle or sensorimotor instability, which delays the postural adjustment that must be made during landing. Repeated movements can develop chronic injuries if they are not properly treated and usually occur due to inadequate dynamic stabilization provided by the skeletal musculature. Acrobatics generate overload on the structures that will support the body weight when they return to the ground, which can generate major impacts on the knee joints [13], ankles, and shoulders. As mentioned above, although capoeira is a non-contact sport, contact can occur if a practitioner is unable to dodge the blow efficiently.

Regarding the maneuver that caused the most injury in this study, the armada (9%), martelo (8%), ginga (5%), mortal (5%), parafuso (5%), and tesoura (5%) were the most mentioned by athletes. The armada consists of a kick with the outside of the foot, in which one foot is firmly on the ground and the body rotates 360° behind, placing one’s back to the opponent, and the kick is given with the limb that is not on the ground; the kick is supported and is aimed to hit the opponent’s head. The martelo consists of a kick with the instep of the foot and can be one of three types: standing martelo (which performs a 90° flexion of the hip and knee with a body turn and then performs the kick with a knee extension), martelo-on-the-ground (which consists of placing one hand on the ground next to the torso and raising the opposite lower limb to hit the opponent by turning the body), and hammer-rotated (which is same movement as the martelo-on-the-foot, only that the practitioner continues the 360° turn of the body until the initial position). The ginga is the basic movement of capoeira that consists of a repetitive movement of putting the right hand forward and the right foot back, then doing the same with the left side of the body. It is from this basic movement that all other capoeira moves begin. The mortal consists of a jump that can be forwards or backwards, as if the athlete was going to perform a somersault in the air. Performing this gesture presents a high risk of falling and the possibility of sprains when the feet hit the ground in addition to generating a high impact on joint structures. The parafuso performs a turn that is similar to that of the armada, but when the lower limb begins to turn, the practitioner jumps and performs a side kick with the other limb, spinning in the air. In the tesoura maneuver, the practitioner rests their hands on the ground and places one of their lower limbs behind the opponent’s thigh; the practitioner places the other limb in front of the opponent’s abdomen and, as if they were scissors, rotates the body in order to overthrow the opponent.

All of the maneuvers mentioned above involve a lot of rotations, both at the spine and at the knee, with a high speed force being applied when applying the blow; they require good sensorimotor and ligament stability in the case of the knees and, when considering the spine, the rotation movements promote greater stress on the intervertebral discs and may lead to dehydration and premature aging of the discs [25]. Some of these maneuvers involve jumps that need muscle power to perform them and a high activation of the neuromuscular spindles with an adequate functioning of the stretching–contraction cycle in addition to generating a great impact on the joints when the feet or hands are received on the ground, which requires great osteo-mio-ligamentous and sensorimotor stability.

Regarding treatment, most injuries were treated with rest (28%), physiotherapy (23%), and medication (20.9%). In the study by Freire et al. [18], 25% of the athletes used some medication to treat the injuries, 23.6% sought a doctor, 16.7% used ice on the injured sites, and about 15% underwent physiotherapy. In the study by Zucca and Grüninger [13], most athletes went to the doctor (46%), underwent physical therapy (33%), and self-medicated (21%), while 35% of athletes did not seek help treating their injuries.

Although most of the injuries were submitted to some type of intervention in our study, most of the athletes in the sample (39%) did not stop practicing capoeira because of the injury, despite practicing it conditionally, whereas 19% of the athletes in the sample stopped practicing capoeira for a period of longer than 30 days. In the study by Zucca and Grüninger [13], 77% of practitioners needed to leave training, reduce the training pace, and/or needed a health professional due to the injury. In the study by Freire et al. [18], 42% of athletes recovered from injuries in less than a month and approximately 27% recovered in the period ranging between 1 and 2 months; it was reported that 39% referred to a loss of physical conditioning.

Most of the injuries in our sample (49%) were fully recovered, and the athletes no longer had pain or other symptoms; however, data from the study by Freire et al. [18] revealed that 51% of athletes still feel pain due to injuries.

The data obtained by binary logistic regression revealed that men were 2.19 times more likely to develop injuries compared with women. Women have certain physiological characteristics, particularly hormonal variations caused by estrogen levels, that influence structures in connective, ligament, and muscle tissue, which provides greater laxity in these tissues [26]; in the case of capoeira, which involves movements that require flexibility, this could be considered a protective factor against injuries for women.

Athletes who trained three or more times per week were more likely to develop injuries compared with those who trained up to two times per week. Excessive capoeira training without specific muscle and sensorimotor reinforcement training can lead to the overloading of structures that do not have adequate stability and strength.

This study had some limitations, namely the fact that the injuries were reported by the athletes themselves and were dependent on memory bias, as well as the fact that other factors may have influenced or exacerbated the injury, such as the practice of another sport. Nevertheless, it is necessary to carry out future studies that use records of musculoskeletal injuries that are diagnosed by health professionals. It would also be interesting to carry out future studies that evaluate the intensity of capoeira training in order to verify its relationship with the presence of injury.

## 5. Conclusions

The data of this study showed a high prevalence of injuries in this analyzed stratified sample, with the ankles, knees, shoulders, and lumbar spines being the most injured body areas and sprains, muscle bruises, and ligament injuries being the more prevalent type of injury. Regarding the injury mechanisms, falls, repetitive movements, acrobatic heels, and contact with another athlete were the most prevalent, and the armada and martelo maneuvers were the maneuvers where injury was more frequent. The risk factors that were associated with the presence of injuries were sex (male) and a training frequency that was equal to or greater than three times per week.

Capoeira has significant representation in Brazilian culture and in the world, and it is currently considered as an international popular cultural phenomenon, originally being an art that came out of marginalization and prohibition by the penal code (1890) that is now registered as Brazilian cultural heritage. Studies on this modality and studies that are representative of it are still scarce. Regardless, this type of study becomes necessary so that it can be used to create specific training programs with the objective of preventing injuries on capoeira athletes, reducing the time lost from training due to the athlete’s absence, reducing health expenses with the treatment of injuries, and promoting the improvement of the athlete’s performance.

## Figures and Tables

**Table 1:** Type and location of injury.

Type of Injury	Location of Injury	*n*	%
Fracture	Arm	2	
	Wrist	1	
	Pelvis	1	
	Foot/Toes	3	
	Thorax/chest/ribs	1	
	All	8	10.26
Muscle bruise	Face/cranium	3	
	Shoulder	1	
	Knee	1	
	Leg	3	
	Ankle	1	
	Foot/Toes	1	
	Lumbar spine	1	
	All	11	14.10
Muscle sprain	Wrist	1	
	Arm	1	
	Forearm	1	
	Knee	1	
	Leg	1	
	Cervical spine	1	
	All	6	7.69
Muscle rupture	Arm	1	
	Thigh	2	
	Leg	2	
	All	5	6.41
Meniscal/disc injury	Knee	3	
	Cervical spine	1	
	All	4	5.13
Cartilage damage	Knee	1	
	All	1	1.28
Sprain	Wrist	1	
	Knee	4	
	Ankle	10	
	All	15	19.23
Tendinopathy	Wrist	1	
	Shoulder	2	
	Ankle	1	
	All	4	5.13
Ligament injury	Wrist	1	
	Shoulder	3	
	Knee	2	
	Ankle	3	
	All	9	11.54
Luxation	Elbow	1	
	Ankle	1	
	All	2	2.56
Low back pain	Lumbar spine	6	
	All	6	7.69
Neck pain	Cervical spine	1	
	All	1	1.28
Non-specific pain	Thorax	1	
	Wrist	1	
	Shoulder	1	
	All	3	3.85
Osteoarthritis (with prosthesis placement)	Hip	1	
	All	1	1.28
Osteoarthritis (without prosthesis placement)	Knee	1	
	Hip	1	
	All	2	2.56
TOTAL	78		100

**Table 2:** Mechanisms of injuries.

Injury Mechanisms	*n* (%)
Fall	19 (24.36%)
Repetitive movement	12 (15.38%)
Acrobatic heels	9 (11.54%)
Contact with another athlete	9 (11.54%)
Twist movement	6 (7.69%)
Landing	4 (5.13%)
Doddy	4 (5.13%)
When supporting the weight with one hand	2 (2.56%)
Blow (kick)	1 (1.28%)
Change in direction	1 (1.28%)
The athlete could not answer	11 (14.10%)

**Table 3:** Injury maneuvers.

	*n* (%)
Armada	7 (8.97%)
Martelo	6 (7.69%)
Ginga	4 (5.13%)
Mortal	4 (5.13%)
Parafuso	4 (5.13%)
Tesoura	4 (5.13%)
Aú	3 (3.85%)
Queda de 4 apoios	3 (3.85%)
Stretching	2 (2.56%)
Aú de coluna sem as mãos	2 (2.56%)
Ponte/meia lua de compasso	2 (2.56%)
Rasteira	2 (2.56%)
Negativa	2 (2.56%)
Armada dupla	1 (1.28%)
Meia-lua de frente	1 (1.28%)
Benção	1 (1.28%)
Esquiva	1 (1.28%)
Flic para trás	1 (1.28%)
Meia-lua	1 (1.28%)
Mortal Trançado	1 (1.28%)
Queixada	1 (1.28%)
Do not know	20 (25.64%)
Total	78 (100%)

**Table 4:** The relationship between the event, the presence of injury, and variables about non-modifiable sample factors and capoeira practice characteristics.

Variables	OddsRatio_crude_ (CI 95%);	*p*-Value
Sex (female *) male	2.19 (1.05–4.61)	0.037
Age group (up to 19 years old *) ≥ 20 years old	1.31 (0.66–2.56)	0.439
Years of practice (up to 5 years *) ≥ 6 years	1.14 (0.58–2.27)	0.702
Weekly training (up to 2 times *) ≥ 3 times	0.44	≤0.001
Duration of training per week (up to 2 h *) ≥ 3 h	1.34 (0.62–2.89)	0.457
Participation in competitions (no *) yes	3.23 (0.87–11.92)	0.079
Type of capoeira (regional *) contemporary	1.13 (0.49–2.57)	0.772
Graduation (gray, yellow, orange, green *) monitor, instructor, professor, counter master, master	1.45 (0.61–3.42)	0.398
Graduation (gray, yellow, orange, green, monitor, instructor, professor, counter master, master *) raw	1.12 (0.56–2.27)	0.751

* Class reference.

## Data Availability

The data obtained in this study are included in an SPSS database. None of these documents are available online.

## References

[1] Monteiro A., Ennes F., Ugrinowitsch H., Vieira M., Benda R. (2015). Tempo de reação de escolha de capoeiristas iniciantes e experientes. Rev. Bras. Ciênc Esporte.

[2] Lima P., Camelo P., Ferreira V., do Nascimento P., Bezerra M., Almeida G., de Oliveira R. (2018). Evaluation of the isokinetic muscle function, postural control and plantar pressure distribution in capoeira players: A cross-sectional study. Muscles Ligaments Tendons J..

[3] Silva R. (2016). Capoeira em Terra de Alemão: A Invisibilidade Cultural.

[4] Campos J., Dibai-Filho A., Cordeiro M., Mariano E., Souza S. (2021). Disability and pain in capoeira practitioners. Rev. Assoc. Med. Bras..

[5] Federação Portuguesa de Capoeira. https://www.fpcapoeira.org/.

[6] Araujo S., Cohen D., Hayes L. (2015). Six weeks of core stability training improves landing kinetics among female capoeira athletes: A pilot study. J. Hum. Kinet..

[7] Mariconda M., Cozzolino A., Di Pietto F., Ribas M., Bellotti V., Soldati A. (2014). Radiographic findings of femoroacetabular impingement in capoeira players. Knee Surg. Sports Traumatol. Arthrosc..

[8] Delamont S., Ribeiro T., Lloyd I., Stephens N. (2021). Os Joelhos! Os Joelhos! Protective Embodiment and Occasional Injury in Capoeira. Front. Sociol..

[9] Muzenza. https://muzenza.com.br/site/portfolio/livros/.

[10] Cunha I., Vieira L., Tavares L., Sampaio T. (2014). Capoeira: A memória social construída por meio do corpo. Movimento.

[11] Falcão J. (2006). O jogo da capoeira em jogo. Rev. Bras. Cienc. Esporte.

[12] DW Roda de Capoeira é Patrimônio da Humanidade. https://www.dw.com/pt-br/unesco-reconhece-capoeira-como-patrim%C3%B4nio-cultural-imaterial-da-humanidade/a-18090747.

[13] Zucca L., Grüninger B. (2020). Incidence of musculoskeletal injuries in capoeira practitioners. Rev. Intersaúde.

[14] Carvalho R. (2016). Gingando na Lusofonia: A institucionalização da capoeira em Portugal. Rev. Cienc. Soc..

[15] Bonfim G. (2013). The suffer of lesions in practitioners of capoeira at the city of Fortaleza. Rev. Diálogos Acadêmicos.

[16] Neto M., Rosário M., Arcanjo F., Conceição C. (2012). Comparative study of musculoskeletal injuries in different types of capoeira. Rev. Ter. Man..

[17] Moraes E., Silva M., Santos J. (2003). A prevalência de lombalgia em capoeiristas do Rio de Janeiro. Fisioter. Bras..

[18] Freire V., Costa V., Vasconcelos F., Escudeiro S., Machado A. (2015). Prevalência de lesões em praticantes de capoeira da cidade de Fortaleza/CE. Rev. Bras. Prescrição Fisiol. Exerc..

[19] Caine C., Caine D., Linder K. (1996). Epidemiology of Sports Injuries.

[20] Bonita R., Beaglehole R., Kjellström T. (2006). Basic Epidemiology.

[21] Sanchez D., Luiz C., Silva P., Casa Junior A. Prevalência de morbidades musculoesqueléticas referidas em praticantes de capoeira. Proceedings of the Anais do II Congresso Brasileiro e I Congresso Internacional da Associação Brasileira de Fisioterapia Traumato-Ortopédica—ABRAFITO.

[22] Andrade S. (2016). Caracterização de Lesões em Praticantes de Capoeira do Distrito Federal. [Trabalho de Conclusão de Curso Bacharelado em Fisioterapia]. Bachelor’s Thesis.

[23] Signoreti M., Parolina E. (2009). Análise postural em capoeiristas da cidade de São Paulo. Aspectos fisiológicos e biomecânicos. Rev. Fac. Ciências Saúde. Edições Univ. Fernando Pessoa.

[24] Carneiro R. (2017). Capoeira—Aptidão Física e Lesões: Um Guia para o Treinamento Seguro e Eficaz.

[25] Kapandji A. (2008). Fisiologia Articular: Esquemas Comentados de Mecânica Humana.

[26] VanPutte C., Regan J., Russo A., Seeley R., Stephens T., Tate P. (2016). Anatomia e Fisiologia de Seeley.

